# Spontaneous immortalization of mouse liver sinusoidal endothelial cells

**DOI:** 10.3892/ijmm.2015.2067

**Published:** 2015-01-13

**Authors:** XIUHUA ZHAO, QIAN ZHAO, ZHEN LUO, YAN YU, NA XIAO, XUAN SUN, LAMEI CHENG

**Affiliations:** 1 Department of Obstetrics and Gynecology, The Second Affiliated Hospital, Medical School of Xi’an Jiaotong University, Xi’an, Shanxi 710004, P.R. China; 2 Department of Adult Stem Cells, Institute of Reproduction and Stem Cell Engineering, Central South University, Changsha, Hunan 410078, P.R. China; 3 Department of Adult Stem Cells, National Engineering and Research Center of Human Stem Cells, Changsha, Hunan 410078, P.R. China

**Keywords:** spontaneous immortalization, liver sinusoidal endothelial cell, proliferation, phenotype

## Abstract

The spontaneous immortalization of cells *in vitro* is a rare event requiring genomic instability, such as alterations in chromosomes and mutations in genes. In the present study, we report a spontaneously immortalized liver sinusoidal endothelial cell (LSEC) line generated from mouse liver. These immortalized LSECs showed typical LSEC characteristics with the structure of transcellular fenestrations, the expression of von Willebrand factor (VWF) and the ability to uptake DiI-acetylated-low density lipoprotein (DiI-Ac-LDL). However, these immortalized LSECs lost the ability to form capillary-like structures, and showed clonal and multilayer growth without contact inhibition. Moreover, their proliferation rate increased with the increase in the number of passages. In addition, these cells obained the expression of CD31 and desmin, and showed an upregulation of p53 protein expression; however, their karyotype was normal, and they could not form colonies in soft agar or tumors in SCID mice. In conclusion, in the present study, we successfully established a spontaneously immortalized LSEC line.

## Introduction

The liver sinusoid is regarded as a unique capillary differing from others (1), and the liver sinusoidal endothelial cells (LSECs) have a typical phenotype, well integrated into the special needs of the liver. LSECs can form a fenestrated barrier between blood and hepatocytes, which permits greater oxygenation for hepatocytes and promotes the more efficient clearance of drugs, perhaps also of chylomicron remnants (2,3). Additionally, the association between LSECs and hepatocytes may be critical for the recovery of hepatocytes from toxic injury (4,5).

Generally, the ability of normal cells to proliferate *in vitro* is tightly controlled. Cells have a finite lifespan, experiencing replicative senescence and eventual death after a certain number of cell divisions (6–8). However, increasing evidence indicates that some types of rodent cells, such as 3T3 fibroblasts, mouse epidermal cells and rat epithelial cells are capable of spontaneous immortalization *in vitro* (9–12). These immortalized cells have emerged from replicative senescence, have lost contact inhibition and have piled up on top of each other to form foci (13). It is believed that genetic instability plays a crucial role in spontaneous immortalization, including alterations in chromosomes and mutations in genes, such as p53 (14–16). However, the molecular mechanisms involved remain obscure.

In the present study, we successfully isolated, purified and cultured LSECs. After a prolonged culture, these LSECs gradually experienced senescence and post-senescence and eventually became immortalized. We further performed a detailed characteristics analysis for these immortalized LSECs. The results indicated that although some distinctive phenotypes were maintained, these immortalized LSECs obtained certain novel biological characteristics which rendered them different from early passage cells.

## Materials and methods

### Preparation of LSECs

The present study was approved by the Ethics Committee of Central South University, Changsha, China. After Kunming white mice (n=6; Central South University Animal Studies) were sacrificed by cervical dislocation, the whole liver was completely resected and repeatedly washed with phosphate-buffered saline (PBS; Gibco, Carlsbad, CA, USA). In order to avoid any potential contamination by large vessel and biliary endothelial cells, identifiable vascular structures were excised from the liver specimens. The remaining liver tissue was sectioned into 5-mm^3^ cubes, and then transferred to a dish containing 2.0 U/ml of dispase and 1X penicillin-phytomycin (Sigma, St. Louis, MO, USA) and incubated at 4°C for 24 h. After terminating the digestion with 10% fetal bovine serum (FBS; Gibco) in MCDB 131 medium (Sigma), the liver cubes were mechanically disaggregated in MCDB 131 medium with a flat instrument to release the endothelial cells. The cell suspension was transferred to a 15-ml conical tube and centrifuged at 600 × g for 10 min. Following centrifugation, the supernatant was discarded and the pellet was resuspended in appropriate volumes of MCDB 131 medium. The cell suspension was then pipetted onto a density gradient of 35% Percoll (Sigma) and centrifuged at 12,000 × g, 4°C for 15 min. Following centrifugation, the band which was located on the red cell band of the gradient was transferred very carefully to a 15-ml conical tube containing PBS. After mixing gently, the sample was centrifuged at 600 × g, 4°C for 10 min and the pellet was resuspended in MCDB 131 medium. Following centrifugation at 100 × g for 5 min, the pellet was suspended in the liver endothelial cell culture medium and plated on 6-well tissue culture dishes pre-coated with fibronectin (Sigma). Non-adherent cells or debris were removed by washing steps after 5 h of culture at 37°C in 5% CO_2_ in a humidified incubator. The adherent cells were further washed with complete endothelial cell selective medium and cultured in the same medium. The endothelial cell selective medium contained 40% MCDB 131, 40% endothelial cell growth medium (EGM)-2 (Lonza, Basel, Switzerland), 10% FBS and 10% endothelial cell conditioned medium (EC-CM, see below). The medium was also supplemented with the following growth factors: 1% L-glutamine (Gibco), 10 ng/ml vascular endothelial growth factor (VEGF; Invitrogen, Carlsbad, CA, USA), 10 ng/ml basic fibroblast growth factor (bFGF; Invitrogen) and 1 ng/ml dexamethasone (Sigma).

### Preparation of EC-CM

The preparation of the EC-CM was as follows: The mouse bone marrow endothelial cell line (a gift from Professor Qiru Wang, Central South University, China) was cultured in Iscove’s modified Dulbecco’s medium (IMDM) with 10% FBS until 80% confluent. The medium was replaced with 5 ml IMDM without serum in each 100-mm plate to collect the conditioned medium. Following incubation for 24 h, the culture medium was collected. The collected conditioned medium was centrifuged at 740 × g for 20 min. The supernatant was then filtered with a 0.22-*μ*m filter and stored at −20°C until use. EC-CM was used within a week after being thawed.

### Flow cytometry

All staining was performed according to the manufacturer’s instructions, using 10^6^ cells and the recommended amount of antibodies, followed by incubation for 30 min at room temperature. Briefly, the cells were incubated separately with the primary antibody rat anti-mouse CD31 (ab56299, 1:50; Abcam, Burlingame, CA, USA) for 30 min. After washing 3 times with PBS, the cells were incubated with the secondary antibody goat anti-rat Alexa Flour 488 (A-11006, 1:1,000; Invitrogen). For the negative controls, the cells were incubated only with the secondary antibody. The expression level of CD31 was determined using a FACScan flow cytometer (BD Biosciences, Franklin Lakes, NJ, USA).

### Immunoperoxidase and immunofluorescence staining

The LSECs were seeded in fibronectin-coated coverslips. The cells were fixed in 4% paraformaldehyde for 15 min. After washing 3 times with PBS, the cells were incubated for 1 h at room temperature in PBS containing 5% BSA (Sigma) to block non-specific binding. For determining the expression of von Willebrand Factor (vWF), the cells were premeabilized by incubation with 0.5% Triton X-100 (Sigma) in PBS for 10 min at room temperature and then incubated with mouse anti-mouse vWF antibody (555849, 1:200; BD Biosciences) followed by incubation with avidin-biotin complex (EastCoast Bio, Birmingham, UK) for 60 min at room temperature. The peroxidase reaction was developed with 0.3 mg/ml 3-3′-diaminobenzidine (EastCoast Bio) in buffer containing 0.05% hydrogen peroxide (EastCoast Bio) for 5–10 min at room temperature. Images were obtained under a phase contrast microscope (Nikon, Tokyo, Japan). For determining the expression of other CD genes, the cells were incubated with primary antibodies [rat anti-mouse CD31 (ab56299), rat anti-mouse CD105 (ab188488), mouse anti-mouse CD90 (ab226), goat anti-mouse CK19 and goat anti-mouse desmin (ab80503); Abcam] at a 1:50 concentration followed by incubation with corresponding secondary antibodies of goat anti-rat Alexa Flour 488 (A-11006, 1:1,000; Invitrogen), rat anti-mouse Ig-FITC (RMG101, 1:250; Invitrogen) and bovine anti-goat Ig-FITC (1:250; Invitrogen) for 30 min at room temperature. The nuclei were counterstained with 4′,6-diamidino-2-phenylindole (DAPI; Sigma), and the slides were mounted with vector-shield, covered and sealed with nail polish. Images were acquired under an inverted fluorescent microscope (Nikon).

### Uptake of DiI-acetylated-low density lipoprotein (DiI-Ac- LDL)

An incorporation analysis of DiI-Ac-LDL was performed. The cell monolayer was incubated with 10 *μ*g/ml of DiI-Ac-LDL (Biomedical Technologies Inc., Stoughton, MA, USA) in medium for 4 h at 37°C, washed 3 times and examined for the uptake of DiI-Ac-LDL under an inverted fluorescence microscope (Nikon).

### Capillary tube formation assay

The cells (2×10^4^) suspended in 200 *μ*l EGM-2 medium supplemented with 50 ng/ml VEGF and 1% FBS were plated onto 50 *μ*l of Matrigel (BD Biosciences) in a 96-well plate. Endothelial progenitor cells (EPCs) were used as a positive control, which were derived from the cord blood as previously described (40). The degree of tube formation was observed and photographed every 2 h under an inverted phase contrast microscope (Nikon).

### Scanning electron microscopy

The cells were fixed in 2.5% glutaraldehyde in 0.1 M cacodylate buffer (Sigma) pH 7.4 for 6 h. After rinsing with ddH_2_0, the specimens were post-fixed with 1% OsO_4_ (Sigma) in ddH_2_0 for 1 h, then rinsed with ddH_2_0 prior to dehydration in a series of graded ethanol solutions and subsequently embedded in a mixture of Epon-Araldite. Thin sections were cut using a diamond knife mounted on an LKB Ultratome and stained with aqueous uranylacetate. The specimens were examined under a JEOL 1200 EX electron microscope (Jeol, Tokyo, Japan).

### Senescence-associated β-galactosidase (SA-β-gal) assay

Positive SA-β-gal staining has been reported to reflect the replicative senescence of cells, but not in quiescent or terminally differentiated cells, and the stained cells are representative of the enzymatic activity (17). SA-β-galactosidase staining was performed as previously described (16). Briefly, the cells were fixed with 0.5% glutaraldehyde (pH 7.2). After washing with PBS (pH 7.2) supplemented with 1 mM MgCl_2_, the cells were stained in X-gal solution [1 mg/ml X-gal, 0.12 mM K_3_Fe (CN)_6_, 0.12 mM K_4_Fe (CN)_6_, 1 mM MgCl_2_] in PBS at pH 6.0 overnight at 37°C. The senescent (stained) and non-senescent (unstained) cells were viewed under a microscope (Nikon).

### Growth characteristics of immortalized LSECs

The density of the immortalized LSEC suspension at passages 20 and 21 was adjusted to 3×10^3^ cells/ml and plated on 24-well tissue culture dishes. Every other day, the cells were digested and enumerated. Cell growth kinetic curves were generated and the doubling-time was cacluated using the following formula: T = 0.693 (T2 − T1)/ln (N2 − N1) where T2−T1 is the difference in the value of time of twice successive detections; and N2−N1 is the difference in the value of cell numbers of twice successive detections.

### Western blot analysis

Cell extracts were prepared using radio-immunoprecipitation assay lysis buffer (Sigma) containing 1 mM phenylmethanesulfonyl fluoride (Sigma). Proteins (50 *μ*g) in the cell extracts were separated on a 10% SDS-PAGE gradient (Invitrogen) and transferred onto PVDF membranes (Millipore, Billerica, MA, USA) in accordance to the manufacturer’s instructions (Invitrogen). The membranes, which had been blocked with 5% skim milk, were incubated with mouse anti-p53 antibody (sc-374087, 1:500; Santa Cruz Biotechnology, Santa Cruz, CA, USA) and mouse anti-β-actin antibody (A1978, 1:3,000; Sigma), followed by incubation with horseradish peroxidase-conjugated goat anti-mouse IgG secondary antibody (ab175740, 1:10,000; Abcam). After washing with PBS-T solution 4 times, the immunoreactive bands were detected by electrochemiluminescence (Sigma) by exposing the blots.

### Cytogenetics analysis

The cells were harvested and G-banded chromosome preparations were made using standard methods (18). The harvested cells were fixed in a methanol:acetic acid (3:1) solution. The chromosomes were stained with 4% Giemsa solution in Gurr buffer and the number and G-C band of the chromosomes in metaphase from each cell line were determined.

### Soft agar assay

To measure cell anchorage independence, the cells (1×10^4^) were plated in 6-well soft agar dishes (0.5% bottom and 0.33% top agar, respectively) for 3 weeks. HPG-2 cells (human hepatocellular carcinoma cell line; Cell Bank of Central South University, Changsha, China) were used as positive controls. The ability for anchorage-independent growth shown by colony formation was analyzed after 20 days. Colonies containing >10 viable cells were scored as positive.

### Tumorigenesis in SCID mice

The immortalized LSECs were assayed for the ability to form tumors in SCID mice. Ten million viable cells were injected into the lower limb muscles of 6- to 8-week old SCID mice. Control mice were injected with HPG-2 cells. The animals were observed at regular intervals for tumor development for a period of 6 months. The mouse muscle tissue sections were stained with hematoxylin and eosin (H&E).

## Results

### Isolation, culture and phenotype characteristics of LSECs

**Figure 1:**
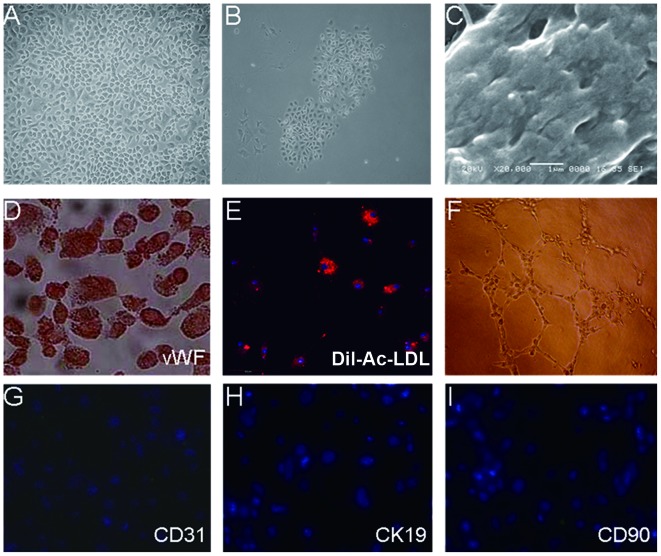
Characterization of liver sinusoidal endothelial cells (LSECs). (A) Morphology of LSEC monolayer showing the typical cobblestone morphology characteristics of endothelial cells (mangification, x40); (B) morphology of sinusoidal endothelial cell colonies; (C) scanning electron microscopy of LSECs showing the typical round fenestrae on cell surface (scale bar, 1 *μ*m); (D) LSECs showing positive staining for von Willebrand factor (vWF, red; magnification, x200); (E) uptake of DiI-acetylated-low density lipoprotein (DiI-Ac-LDL; red). (F) LSECs formed capillary-like structures in Matrigel after 14 h of culture; (G-I) represented negative staining for CD31, CK19 and CD90, respectively (antibody, green; DAPI, blue; magnification, x200).

After the plating of the cells isolated from the mouse livers on fibronectin-coated culture dishes, small adherent clusters of cells were observed within 24 h. With the prolonged culture, these clusters slowly became enlarged until they formed a confluent monolayer. Replating of the cells after digestion revealed clonal growth and a typical cobblestone morphology, a characteristic of endothelial cells (Fig. 1A); the colonies were picked up, pooled and expanded for several weeks, and these culture showed a very homogenous morphology and strictly grew as a monolayer (Fig. 1B). The contaminating non-endothelial-like cells were successfully eliminated by the use of endothelial-selective medium and contrast-digestion after the second passage in serial culture.

At present, the only gold standard defining LSECs is the presence of fenestration with a diameter of 100–150 nm. We examined the monolayer of the cultured LSECs by transmission electron microscopy. As shown in Fig. 1C, these cells had typical transcellular fenestrations.

The most commonly used characteristics to identify endothelial cells include the expression of vWF and CD31, the uptake of acetylated low-density lipoprotein (Ac-LDL) and the formation of a capillary-like structure *in vitro* (19,20). The monolayers of the LSECs showed positive staining for vWF expression (Fig. 1D) and the uptake of Dil-Ac-LDL following 4 h of exposure to Dil-Ac-LDL (Fig. 1E). In addition, the LSECs formed typical capillary-like structures in Matrigel after 14 h of culture (Fig. 1F), and the expression of CD31, a marker of vascular endothelial cells, was not detected (Fig. 1G). To examine whether these LSECs were contaminated by other liver cells, we further examined the expression of CK19 (a marker of hepatocytes) and CD105, CD90 and desmin (markers of mesenchymal fibroblast-like cells which reside in perisinusoidal, portal and around the centrilobular vein; desmin is also a classical stellate cell maker). As shown in Fig. 1H and I, the expression of CK19/CD90/CD105/desmin was not detected in the monolayer of LSECs (CD105 and desmin; data not shown).

### Spontaneous immortalization of LSECs

**Figure 2:**
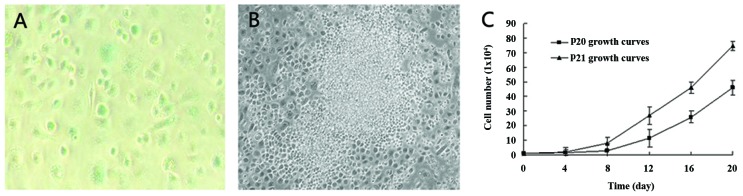
Senescence-associated β-galactosidase (SA-β-gal) staining of senescent liver sinusoidal endothelial cells (LSECs) and the morphology and growth rates of the immortalized sinusoidal endothelia cells. (A) Senescent LSECs were positive for SA-β-gal staining (blue; magnification, x200). (B) The immortalized sinusoidal endothelia cells lost growth inhibition, and showed clonal and multilayer growth. (C) Growth curves of the immortalized sinusoidal endothelial cells at passages 20 and 21 (P20 and P21).

By passage 8, the majority of the LSECs appeared senescent as judged by the presence of numerous LSECs which exhibited a large and flattened morphology and SA-β-Gal activity (Fig. 2A). Following extended culture, a small population of round strong refractive proliferating cells emerged from the senescent cultures, and these cells showed clonal and multilayer growth (Fig. 2B), and their proliferation rate increased with the increase in the number of passages. As shown in Fig. 2C, at passages 20 and 21, these cells continued vigorous growth. The doubling time of the cells at passages 20 was 4.01 days, while the doubling time of the cells at passage 21 was 3.50 days. Thus, the cells at passage 21 displayed a higher proliferation potential than those at passage 20. All these characteristics were consistent with those of immortalized cells (13,14).

### Biological characteristics of the immortalized LSECs

**Figure 3:**
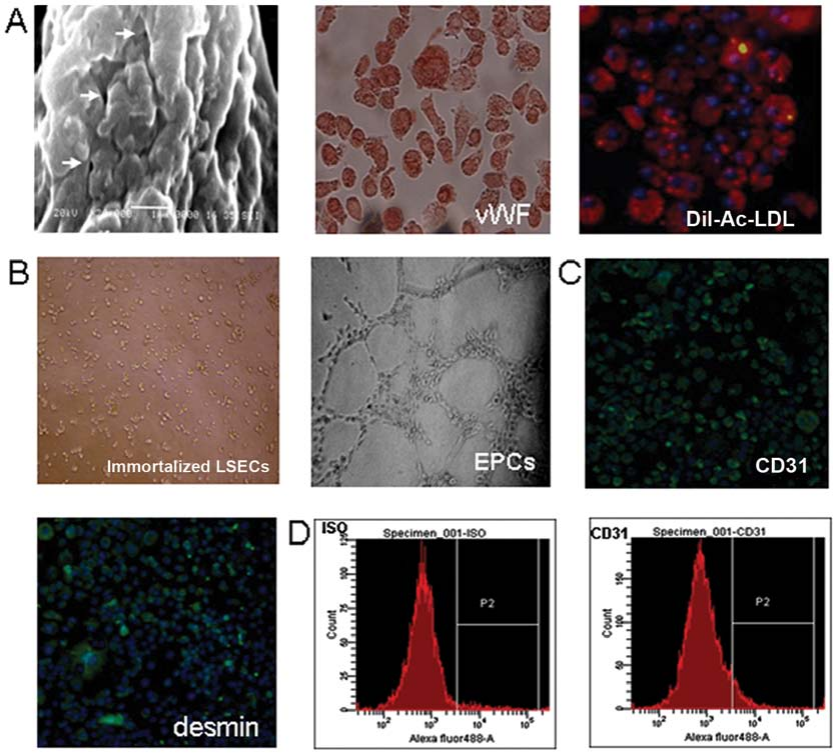
Biological characteristics of the immortalized liver sinusoidal endothelial cells (LSECs). (A) These immortalized LSECs maintained typical fenestration structures (indicated by arrows) on the cell surface (scale bar, 1 *μ*m), the expression of von Willebrand factor (vWF, red; magnification, x200) and the uptake of DiI-acetylated-low density lipoprotein (DiI-Ac-LDL, red; magnification, x200). (B) The immortalized LSECs did not show migration and sprouting after 14 h of incubation, while the endothelial progenitor cells (EPCs) derived from cord blood [as previously described (40)], as a positive control, formed typical capillary-like structures after only 4-h incubation in Matrigel. (C) The immortalized LSECs expressed CD31 and desmin (antibody, green; DAPI, blue; magnification, x200). (D) FACS quantitative analysis showed that the positive percentage of CD31 was 84.1% in the immortalized sinusoidal endothelial cells at passage 15.

To clarify whether the immortalized LSECs had different properties compared to primary LSECs, we examined the characteristics of the immortalized cells. As shown in Fig. 3A, the immortalized LSECs maintained typical transcellular fenestrations, as well as the expression of vWF and the uptake of DiI-Ac-LDL; these characteristics were consistent with those of the primary cells. However, as shown in Fig. 3B, the immortalized LSECs lost the ability to form capillary-like structures, but obtained the expression of CD31 and desmin (Fig. 3C and D).

### Analysis of p53 protein, karyotype and transformed phenotype

**Figure 4:**
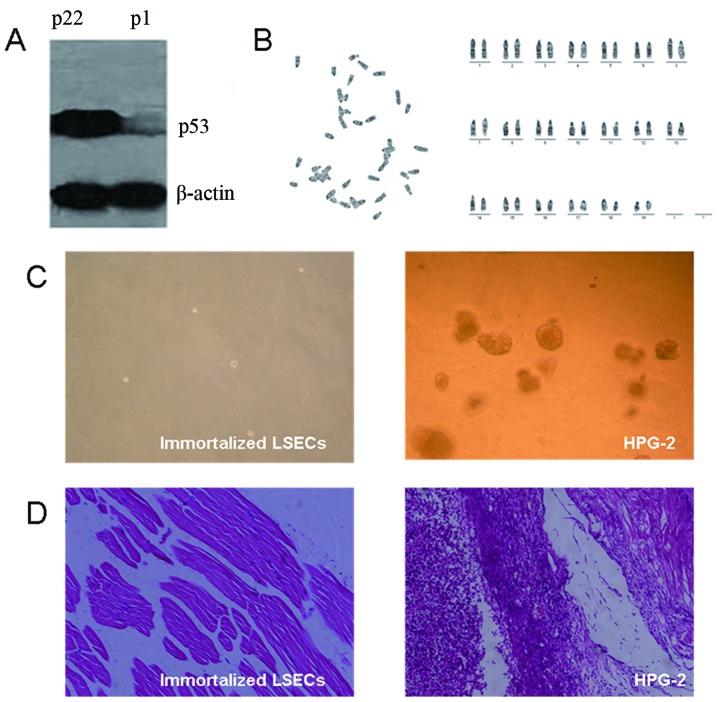
Protein expression of p53, karyotype analysis and tumorigenic potential of immortalized liver sinusoidal endothelial cells (LSECs). (A) The immortalized LSECs expressed high levels of p53 protein. (B) The immortalized LSECs showed a normal karyotype. (C) The immortalized LSECs did not grow in soft agar, in contrast to the HGP-2 cells, which generated colonies. (D) Tumors were not detected in the mouse muscle tissue sections following implantation with immortalized LSECs within 6 months; by contrast, SCID mice implanted with HPG-2 cells developed tumors within 3 weeks (H&E staining).

We then examined p53 protein expression and karyotype in the immortalized LSECs, which are both potentially important factors of immortalization. Compared with the primary LSECs, the protein expression level of p53 in the immortalized LSECs was significantly upregulated (Fig. 4A). However, the immortalized LSECs showed a normal karyotype (Fig. 4B).

Given that these immortalized LSECs showed some characteristics of transformed cells, such as growth characteristics, immunophenotypes and an extended lifespan, we futher analyzed their tumorigenic potential. As shown in Fig. 4C, no colony formation was observed in the immortalized LSECs in comparison to the HPG-2 cells, which served as a positive control. Therefore, the immortalized LSECs were not able to grow in an anchorage-independent manner, indicating no tumorigenic potential and a high differentiation status. This notion was further supported by the fact that all the SCID mice implanted with the immortalized LSECs did not develop any tumors within 6 months; by contrast, the SCID mice implanted with the HPG-2 cells developed tumors within 3 weeks (Fig. 4D).

## Discussion

LSECs are a valuable tool for the study of the physiology and pathophysiology of the liver. Several methods can be used to isolate LSECs, such as the perfusion of an intact organ with an enzymatic cocktail and the mechanical disruption of the organ followed by enzymatic digestion. The cell suspension is then fractionated by differential centrifugation techniques, such as counter flow elutriation or density gradient centrifugation (21–24). In the present study, we isolated LSECs from mouse liver tissue by a combination of mechanical disruption and density gradient centrifugation. Subsequently, these isolated LSECs were further purified based on their different adhesive abilities from other contaminating cells, as well as by the use of endothelial cell selective medium. The endothelial cell selective medium contained EGM-2, MCDB-131, EC-CM and some growth factors, which stimulate the proliferation of LSECs and suppress the growth of Kupffer cells, stellate cells and mesenchymal fibroblast-like cells. A previous study demonstrated that AcSDKP in a <3 kDa fraction of EC-CM had a significant inhibitory effect on the growth of mesenchymal fibroblast-like cells (25). In the process of LSEC culture, mesenchymal fibroblast-like cells were the major contaminating cells, and the addition of EC-CM can efficiently inhibit the growth of mesenchymal stem cells and promote the proliferation of LSECs (26). This method was easy to operate and required no special equipment. Following purification, we finally obtained the homogeneous cultures of LSECs.

In order to confirm that these cells were purified LSECs, we analyzed their structural characteristics, immunophenotypes and other biological characteristics. Under a scanning electron microscope, these cells exhibited a typical fenestration structure on the cell surface. Fenestration is generally considered to be the only gold standard of LSECs, making them clearly distinguishable from all other types of liver cells (27,28). Their endothelial origin was further confirmed by the expression of vWF, the uptake of DiI-Ac-LDL and the formation of capillary-like structures in Matrigel. These characteristics can be used as markers to effectively distinguish LSECs from other types of cells in the liver, including stellar cells and Kupffer cells. During adherent culture, stellate cells exhibit a fibroblast-like morphology. In addition, stellate cells often contain fat and express desmin, but do not express vWF and cannot uptake DiI-Ac-LDL, characteristics which are different from LSECs. Kupffer cells are a type of macrophages in the liver, and they always have many pseudopodia and microvilli, and hence exhibit an irregular morphology. Furthermore, these cells we obtained did not express CD31 (a marker of endothelial progenitor cells and vascular endothelial cells), CK19 (a marker of liver epithelial cells) and CD90, CD105 and desmin (markers of mesenchymal fibroblast-like cells). Hence, we concluded that these cells were purified LSECs which were not contaminated by any other cells in the liver.

It has been demonstrated that immortalized cell lines can generally be established by several methods, such as transgenic technology, adding chemical carcinogen and using γ-ray irradiation (29,30). However, the probability of spontaneous immortalization is extremely low (31). In the present study, LSECs at passage 8 experienced senescence as judged by the phenomenon that numerous LSECs exhibited a large and flattened morphology, and showed SA-β-Gal activity. During extended culture, a small population of round strong refractive proliferating cells emerged from the senescent cultures, and showed clonal and multilayer growth. All these characteristics were consistent with those of the spontaneously immortalized cells reported by other studies (13,14). In the present study, these immortalized LSECs showed some altered characteristics which differed from the early passage LSECs. Although the typical fenestration structure, the expression of vWF and the uptake of DiI-Ac-LDL were maintained in the immortalized LSECs, they lost the ability to form capillary-like structures, and obtained some immunophenotypes, such as the expression of CD31 and desmin. Similar findings have also been reported in other types of cells during spontaneous immortalization (32).

Although the molecular mechanisms of spontaneous immortalization remain unclear, increasing evidence indicates that uncontrolled cellular growth by the activation of oncogenes, the inhibition of cancer suppressor genes and the upregulated activity of telomerase may play crucial roles in the process of immortalization (33,34). It has been reported that a mutation in anti-oncogene p53 leads to its inactivation which may contribute to the spontaneous immortalization of breast epithelial cells during *in vitro* culture (33), and that mutant p53 is only a facilitator of ‘classical’ immortalization in cells with shortened telomeres (34). However, in the present study, p53 in the immortalized LSECs was not inactivated but upregulated. Furthermore, these immortalized LSECs did not show any chromosomal changes or tumorigenic potential *in vitro* and *in vivo*. Similar findings have also been reported in the establishment of a spontaneously immortalized bovine mammary epithelial cell line (35). Based on these results, we speculated that a ‘non-classical’ immortalization may exist, consistent with previous studies demonstrating that the spontaneous immortalization of mouse myogenic cells can occur without the loss of p53 (36) or karyotype abnormality (35).

Previous studies (37,38) have demonstrated that environmental conditions may be relevant to spontaneous immortalization. In the present study, we used endothelial selective condition medium containing EGM-2, MCDB131 and EC-CM derived from immortalized mouse bone marrow endothelial cell lines. It has been reported that mouse bone marrow endothelial cells secrete many types of cytokines, such as granulocyte-macrophage colony-stimulating factor (GM-CSF), interleukin (IL)-6, IL-11 and IL-13 to promote the proliferation of hematopoietic cells (39). Hence, we speculated that the culture conditions for LSECs and long-term subculture may contribute to the immortalization of LSECs.

In conclusion, in this study, we successfully established a spontaneously immortalized mouse LSEC line. These immortalized LSECs maintained some distinctive phenotypes of early passage LSECs, but also obtained certain novel biological characteristics.
